# Application of near-infrared hyperspectral imaging to identify a variety of silage maize seeds and common maize seeds†

**DOI:** 10.1039/c9ra11047j

**Published:** 2020-03-23

**Authors:** Xiulin Bai, Chu Zhang, Qinlin Xiao, Yong He, Yidan Bao

**Affiliations:** College of Biosystems Engineering and Food Science, Zhejiang University Hangzhou 310058 China yhe@zju.edu.cn ydbao@zju.edu.cn +86-571-88982143 +86-571-88982143; Key Laboratory of Spectroscopy Sensing, Ministry of Agriculture and Rural Affairs Hangzhou 310058 China

## Abstract

Common maize seeds and silage maize seeds are similar in appearance and are difficult to identify with the naked eye. Four varieties of common maize seeds and four varieties of silage maize seeds were identified by near-infrared hyperspectral imaging (NIR-HSI) combined with chemometrics. The pixel-wise principal component analysis was used to distinguish the differences among different varieties of maize seeds. The object-wise spectra of each single seed sample were extracted to build classification models. Support vector machine (SVM) and radial basis function neural network (RBFNN) classification models were established using two different classification strategies. First, the maize seeds were directly classified into eight varieties with the prediction accuracy of the SVM model and RBFNN model over 86%. Second, the seeds of silage maize and common maize were firstly classified with the classification accuracy over 88%, then the seeds were classified into four varieties, respectively. The classification accuracy of silage maize seeds was over 98%, and the classification accuracy of common maize seeds was over 97%. The results showed that the varieties of common maize seeds and silage maize seeds could be classified by NIR-HSI combined with chemometrics, which provided an effective means to ensure the purity of maize seeds, especially to isolate common seeds and silage seeds.

## Abstract 2

NIR-HSI and chemometrics were used to identify different varieties of common and silage maize seeds.

## Graphical abstract

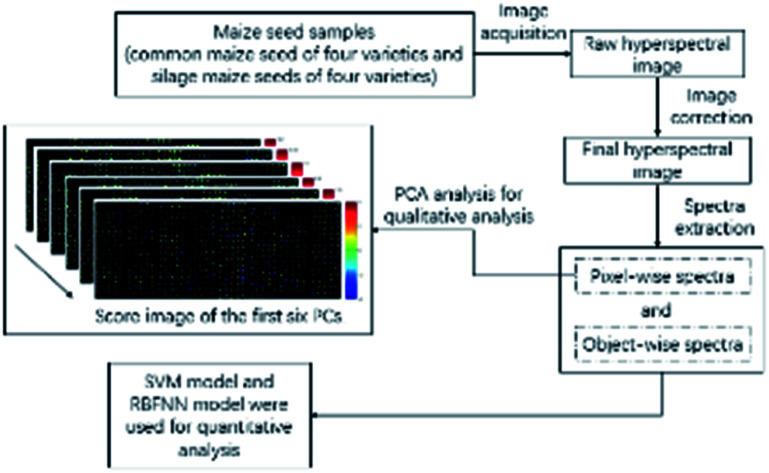

## 1. Introduction

Maize is an important food crop in the world. It can be classified into common (edible) maize and silage maize according to their uses. Common maize is a main food source in our daily life. Common maize has different varieties, including sweet maize, waxy maize and so on. Different varieties have little difference in appearance, but there are differences in yield, quality, nutrition content, *etc.*^1^ Silage maize can also be divided into different varieties according to the suitable growth environment, yield, and nutrient composition of the grain, *etc.*^2^ Compared with common maize, silage maize has the characteristics of high biomass, good fibre quality and good green retention, and is more suitable for animal consumption.^3–5^ Variety purity is an important factor in evaluating seed purity.^6^ Although common maize seeds and silage maize seeds have little difference in appearance, there are differences in internal composition. It is difficult to distinguish a mixture of common maize seeds and silage maize seeds. In the process of maize harvesting and marketing, different varieties of maize seeds are likely to be mixed and are difficult to be detected, which will have a certain impact on sellers and consumers. Strengthening the identification of maize seed purity is the key to ensure the purity of maize seeds. Identification of different varieties of common maize seeds and silage maize seeds is an important step to ensure the purity of maize seeds.

Traditional seed varieties' classification methods include manual inspection, protein electrophoresis, DNA molecular marker technique, *etc.*^7–9^ Manual inspection is mainly based on the external shape (colour, shape, size, *etc.*) of seeds. It is difficult to classify seeds with similar external shape.^10^ Protein electrophoresis technique is based on the content of protein in seeds of different varieties and the speed of protein molecules swimming in the electric field to identify the variety of seeds.^11^ It is accurate and effective, but requires professional operation. It is necessary to extract protein from seeds, which can damage the seeds, and is only suitable for the detection of small samples. For DNA molecular marker technique, a standard DNA fingerprint of seeds must be constructed first. DNA is the main genetic material. It is very accurate and reliable for the classification of the variety of seeds. However, this process requires more financial and material resources, and the establishment of a complete DNA molecular marker identification system requires professional technicians and funds.^12^ It is necessary to establish a rapid, non-destructive and convenient method to classify seed varieties.

With the development of technology, NIR and imaging technique have been applied in seed variety identification. The NIR spectral region is related to the combined frequency and doubled absorption of the vibration of hydrogen-containing groups (such as C–H, N–H and O–H) in organic molecules. Relevant research showed that the spectral reflectance of seeds of different varieties were different, the detection process was fast and convenient, and it was necessary to combine chemometrics to classify seeds of different varieties.^13–16^ Imaging technique has the advantages of nondestructive and convenient operation, and machine vision is widely used in seed variety identification. Through the analysis of colour, shape, texture and other information in the sample images obtained by machine vision technique, it was possible to identify the tiny features that were difficult to be distinguished by the naked eye.^17,18^ However, machine vision only obtains the two-dimensional spatial information of samples in visible bands, and the variety of seeds can be determined accurately with more information. Near-infrared hyperspectral imaging (NIR-HSI) is a fast non-destructive detection technique that integrates spectral technique and imaging technique.^19^ NIR-HSI can simultaneously acquire NIR spectra (one-dimensional spectral information) and image information (two-dimensional spatial information).^20^ Each pixel in a hyperspectral image has spectral information. The spectral information of each pixel combined with the corresponding spatial information can realize the visualization of sample features, which can intuitively show the differences among samples. NIR-HSI has been used to classify the variety of seeds.^21–23^ For maize seeds, Williams *et al.*^24^ used NIR-HSI to classify maize kernels of three hardness categories: hard, medium and soft. Yang *et al.*^25^ classified four varieties of waxy corn seeds. Sendin *et al.*^26^ evaluated the application potential of NIR-HSI to grade whole white maize kernels. NIR-HSI could obtain comprehensive information. Compared with traditional methods, the accuracy of NIR-HSI still needed to be improved, but it had certain reliability. In addition, the simplicity of operation, the convenience of detection and non-pretreatment of samples were conducive to the further application of NIR-HSI in the classification of maize seed of different varieties. In fact, research of the classification of the variety of silage maize seed is relatively less.^9^ Moreover, due to the similarity of appearance, silage maize seeds and common maize seeds are easy to be mixed together and difficult to be distinguished. Therefore, we attempted to classify common maize seeds and silage maize seeds by NIR-HSI.

The main purpose of this research was to explore the feasibility of using NIR-HSI to common and silage maize seeds of different varieties. The classification of eight varieties of maize seeds was studied, including four varieties of common maize seeds and four varieties of silage maize seeds. Support vector machine (SVM) and radial basis function neural network (RBFNN) classification models were established to classify the varieties of maize seeds. Considering the influence of different varieties in maize seeds, the classification of common maize seeds and silage maize seeds was studied, and the classification of four varieties of common maize seeds and four varieties of silage maize seeds were studied respectively.

## 2. Materials and methods

### 2.1 Samples and sample preparation

A total of eight varieties of maize seeds (Yunnan Quchen Seed Co. Ltd., Yunnan, China) were used in the experiment. Four varieties of common maize were involved, including Datian387 (DT387), Quchen8 (QC8), Quchen11 (QC11), and Quchen13 (QC13). Four varieties of silage maize were involved, including Quchen9 (QC9), Quchen19 (QC19), Quchen29 (QC29) and Quchen513 (QC513). For each variety of 5100 kernels, all the samples were normal with clean appearance and no visual damage. A total of 40 800 maize seeds were prepared.

### 2.2 Hyperspectral imaging and spectral acquisition

#### 2.2.1 Hyperspectral imaging system

The hyperspectral images of maize seeds were collected by using a hyperspectral imaging system established in the laboratory. The near-infrared spectral range was 874–1734 nm with 256 bands. The spectral resolution of the hyperspectral imaging system is 5 nm. The hyperspectral imaging system consists of an imaging spectrometer (ImSpector N17E; Spectral Imaging Ltd., Oulu, Finland), a 320 × 256 CCD camera (Xeva 992; Xenics Infrared Solutions, Leuven, Belgium) with a camera lens (OLES22; Specim, Spectral Imaging Ltd., Oulu, Finland), and an IRCP0076 electronically controlled mobile platform (Isuzu Optics Corp., Taiwan, China). Two 150 W tungsten halogen lamps (3900 Lightsource, Illumination Technologies Inc., USA) were symmetrically placed on both sides of the lens of Xeva 992 camera as the light source. A black box is used to cover all instruments. When collecting spectra, the black box is closed to ensure dark conditions. A computer is used to control the system with the software (Xenics N17E, Isuzu Optics Corp., Taiwan).

#### 2.2.2 Image acquisition and correction

Image acquisition was performed at room temperature. At the time of collection, maize seeds were placed on a black flat plate and did not overlap with each other. The flat plate was placed on the conveyor belt for scanning. In order to obtain a clear image without distortion, the height between the camera lens and the sample was set to 12.6 cm, the exposure time of the camera was set to 3 ms, and the conveyor belt moved at a constant velocity of 11 mm s^−1^. The total length of the conveyor belt was 400 mm, and the acquisition time of a hyperspectral image was about 36 s. A black flat plate could place 90 maize seeds, so a hyperspectral image could get the information of 90 seeds. Image processing used ENVI 4.6 (ITT Visual Information Solutions, Boulder, Utah, USA) and MATLAB 2015a (The Math Works, Natick, MA, USA).

After acquire the hyperspectral images of the samples, it is necessary to correct images to reduce the influence of dark current. White and black standard reference images are required and acquired under the same experimental condition of the sample's hyperspectral image acquisition. The white standard reference image was obtained by placing a white Teflon bar with a reflectance of about 100% on the sample position. The black standard reference image was obtained by covering the lens with the opaque lens cap with a reflectance of about 0%. The image correction was carried out by following formulas:1
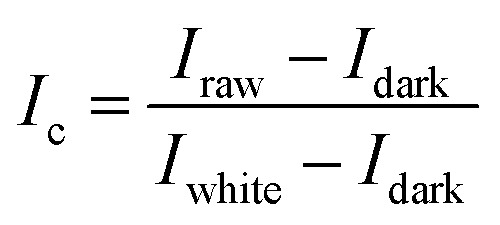
*I*_c_ is the normalized image, *I*_raw_ is the original image, *I*_white_ is the white reference image, and *I*_dark_ is the black reference image.

#### 2.2.3 Spectral data extraction

**Fig. 1:**
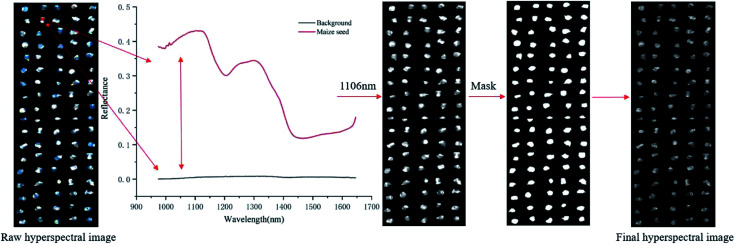
The main steps of spectral extraction.

After correcting the acquired hyperspectral images, the maize seeds and the background need to be separated to extract spectral information of the maize seeds. The entire region of each maize seed was defined as the region of interest (ROI), and 40 800 ROIs were used. As shown in Fig. 1, the reflectance of the maize seed and the background were different, and the highest variance was about at the wavelength of 1106 nm. In this study, the mask was constructed on the image at 1106 nm by setting the pixels of maize seed area to 1 and the pixels of background to 0. The mask was applied to the grayscale image of each wavelength to separate the maize seeds from the background. Then, the spectrum of each pixel in the ROI region was extracted, which was pixel-wise spectra. Wavelet transform of Daubechies 6 with a decomposition level of 3 was used to smooth the extracted pixel-wise spectra for reducing the random noise. Then, the average spectra of each ROI were calculated by averaging the pixel-wise spectra of each ROI. The calculated average spectra were used to represent the corresponding seed sample and regarded as object-wise spectra. Pixel-wise spectra and object-wise spectra were used for analysis. The extraction of the spectra was conducted in MATLAB 2015a (The Math Works, Natick, MA, USA). Due to the influence of optical equipment or surrounding environment, noise of the head and end of the spectra was obvious, so the band with obvious noise was removed and the spectra in the range of 975–1646 nm with 200 bands was used.

### 2.3 Principal component analysis

Principal component analysis (PCA) is to project high-dimensional data into lower-dimensional space. Using a few new variables (principal components (PCs)) to express the data characteristics of original variables as much as possible.^27,28^ Each PC is a linear transformation of the original variable, arranged in descending order of explained variance. The number of PCs can be determined by calculating the cumulative contribution rate of PCs. In this study, the result of PCA analysis showed that the first six PCs reflected 99.98% of the information in the original spectral data. The first six PCs were used to explore the differences among the samples. The loadings of the principal component (PCA loadings) can reflect the correlation between the PCs and the original wavelength variable. The larger loadings of the principal component, the more important the corresponding wavelength variable is. Therefore, the important wavelengths can be recognized. PCA can eliminate the multi-collinearity between variables and reduce data redundancy, and has been applied in near-infrared hyperspectral classification.^29,30^

For hyperspectral images, pixel-wise analysis is a method of visualizing PCA scores.^24^ A single pixel of the image is calculated to obtain a score for each pixel in each principal component hyperspectral image to form a score visualization image. The difference among the samples can be visually observed in the colourmap of each PC.

In this study, PCA was used for qualitative analysis to explore the separability among maize seeds of different varieties. Secondly, PCA loadings was used to recognize important wavelengths to understand the classification process of maize seeds of different varieties.

### 2.4 Classification analysis methods

Support vector machine (SVM) is a generalized linear classifier that classifies data in a supervised learning manner. The raw data is mapped into a high-dimensional space, and the hyperplane with the appropriate boundary is optimized to classify different classes.^31,32^ SVM is a common classification model, which can improve the prediction ability and classification rate by realizing the optimal classification surface. Proper selection of kernel functions is essential to SVM and affects the performance of SVM.^33^ In this study, the radial basis function (RBF) kernel was used to obtain the optimal performance by determining the parameters of penalty coefficient (*c*) and the kernel parameter (*g*). Parameters *c* and *g* were generally determined by grid search method, and their search range were from 2^−8^ to 2^8^.

Radial basis function neural network (RBFNN) is a three-layer forward network.^34^ The first layer is the input layer, which consists of input nodes and does not process information. The second layer is the hidden layer and its number of elements depends on the need to describe the problem. Each neuron in the hidden layer represents a set of radial basis functions. The third layer is the output layer, it responds to the input mode.^35^ The optimal spread value should be determined in the hidden layer. RBFNN can approximate any continuous nonlinear network with arbitrary precision. It has the characteristics of fast learning convergence and simple structure, and has widely used in pattern recognition, function approximation and other fields.^36^ In this study, the RBFNN model for the classification of different varieties of maize seeds were established. The performance and the optimal spread value of the model were evaluated and determined according to the classification accuracy.

SVM and RBFNN were commonly used spectral data analysis models, which could get good analysis results. In this study, SVM and RBFNN models were used to quantify the classification results of the spectra collected using NIR-HSI. At the same time, the classification results of the two models could be compared. It could provide a reference for the development of the application of NIR-HSI to classify common and silage maize seeds of different varieties. The implementation of the SVM and RBFNN model was based on the libSVM and nnet toolbox in MATLAB, respectively.

## 3. Results and discussion

### 3.1 Spectral profile

**Fig. 2:**
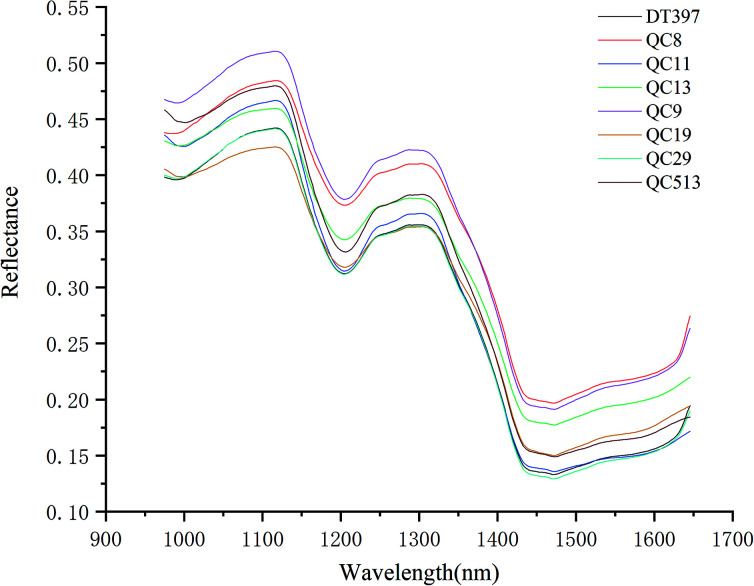
Average spectra of maize seeds of eight varieties in the range of 975–1646 nm.

Using the average spectral reflectance of all pixels of one maize seed represented the spectral reflectance of one maize seed. There were 5100 maize seeds per variety and 5100 spectral curves were obtained for each variety. Due to the obvious noise in the head and end of the spectral curve, the spectra in the range of 975–1646 nm was analysed. The average spectra of eight different varieties of maize seeds was shown in Fig. 2.

According to the spectral curves in Fig. 2, the average spectra of eight varieties of maize seeds had the similar trends. The valley of the spectra at around 1200 nm might be attributed to the second overtone of C–H in carbohydrates.^36,37^ The valley at around 1450 nm was a result of the first overtone of the combination of the C–H bond in the protein and the O–H bond in moisture.^37^ Due to the difference of chemical composition and physicochemical properties among varieties, the spectral reflectance values of different varieties were different, which provided the possibility to classify different varieties of maize seeds. In fact, the overlap among the spectra of maize seeds of different varieties was exist, it was necessary to combine chemometrics methods for further analysis.

### 3.2 PCA scores image visualization

**Fig. 3:**
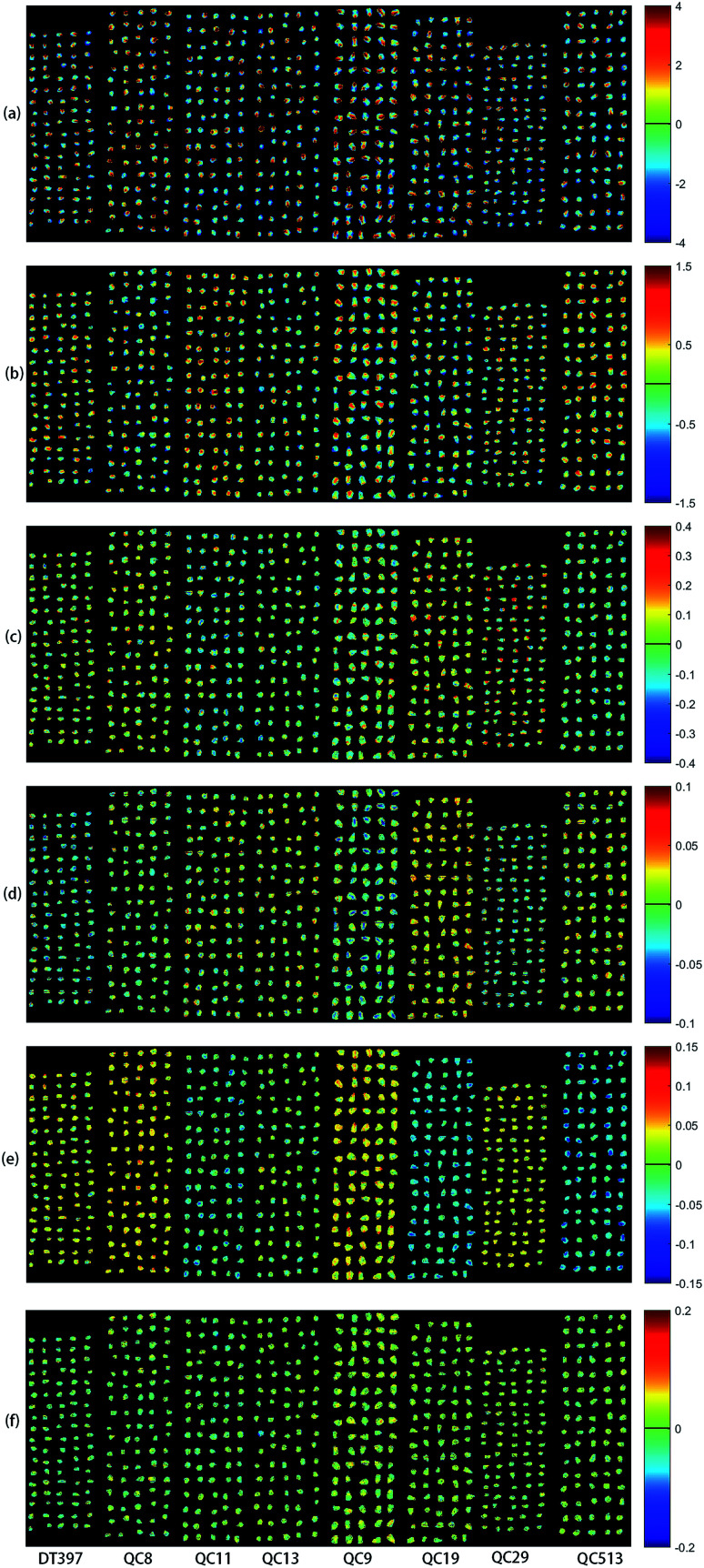
Scores images of the first six PCs of eight maize seed varieties (from left to right: DT397, QC8, QC11, QC13, QC9, QC19, QC29 and QC513): (a) PC1; (b) PC2; (c) PC3; (d) PC4; (e) PC5; (f) PC6.

PCA analysis was performed on the pixel spectral information of eight varieties of maize seeds. A hyperspectral image of each variety was randomly selected in the obtained hyperspectral images for PCA analysis. The result showed that the first six PCs reflected 99.98% of the information in the original spectral data (94.41%, 5.28%, 0.19%, 0.03%, 0.03% and 0.03% for PC1, PC2, PC3, PC4, PC5 and PC6, respectively). That was to say, these six PCs explained most of the variables in the total variance. The scores of the first six PCs were multiplied by the corresponding binary of each pixel in the mask, and the score image was formed and visualized by using the colour bar. Fig. 3 shows the visualized hyperspectral images of the first six PCs of eight varieties of maize seed.

As shown in Fig. 3, the differences among different varieties of maize seeds could be visually displayed by the positive and negative colour scores. It showed that there were differences among different varieties of maize seeds and they could be distinguished. The positive colour scores corresponded warm colours (yellow-red) and negative colour scores corresponded cold colours (green-blue). In score image of PC1, the colour score of hard endosperm of maize seeds was negative, and most of the colour was blue. The colour score of soft endosperm of seeds was positive, and most of the scores were high and the colour was red. In score image of PC2, the colour score of hard endosperm tended to be positive and the colour was green, while the scores of soft endosperm part were still positive and the colour were red and yellow. According to the score images of PC1 and PC2, PC1 and PC2 were mainly colour contrast, which could distinguish between hard endosperm and soft endosperm of maize seeds. Compared with PC1 and PC2, although the contribution rates of PC3, PC4, PC5 and PC6 were relatively small, they could reflect the differences between different varieties of maize seeds. For example, in the score image of PC3, the colour score of the Variety QC9 and Variety QC513 were slightly lower than zero, and the colour score of the Variety QC19 was slightly higher than zero. In the score image of PC4, most of the maize seeds in the Variety DT397 and Variety QC9 had negative colour scores, and the blue colour with larger value proportion appeared. The colour score of variety QC19 was mostly positive with more red appeared. In the score image of PC5, most of the maize seeds in the Variety DT397, Variety QC8, Variety QC9 and Variety QC29 had positive colour scores. Among them, the colour scores of Variety QC8 and Variety QC9 were higher and the colour tended to be yellow. The colour scores of most maize seeds in Variety QC11, Variety QC19 and Variety QC513 were negative, and the colour presented were clearly distinguished from that with positive colour scores. In the score image of PC6, the colour score of Variety QC9 was positive overall with prominent colour presented, and it could be clearly classified. PCA scores images could show the differences among different varieties of maize seeds intuitively, but not all varieties could be distinguished obviously, classification models should be established for further analysis.

**Fig. 4:**
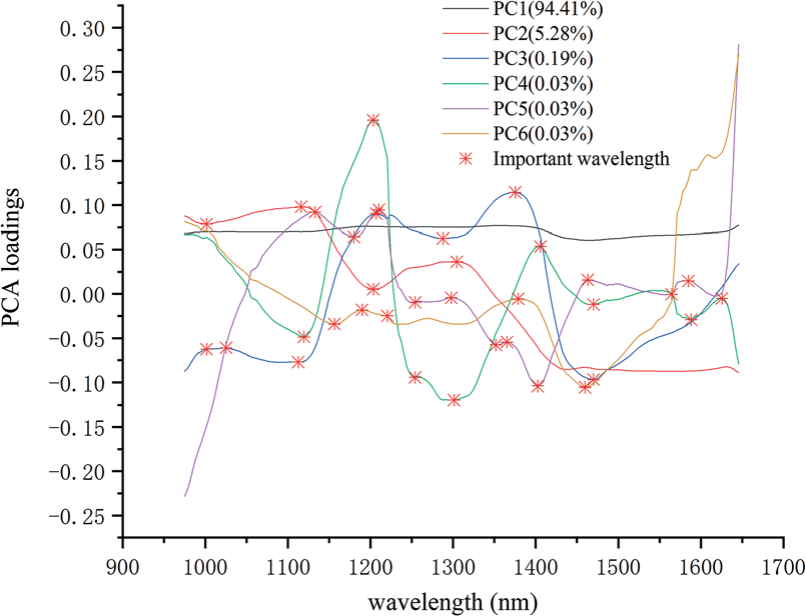
The important wavelengths recognized by PCA loadings.

The important wavelengths were also recognized by using PCA loadings. In the PCA analysis, the cumulative contribution rate of the first six PCs were over 99.98%, so the important wavelengths were recognized by the load of the first six PCs. Fig. 4 shows the wavelength-loading plot for the six PCs. Table 1 shows the important wavelengths recognized by PCA loadings, with a total of 18 important wavelengths. Compared with the full wavelengths, the recognized important wavelengths were corresponded to the chemical composition of maize seeds, which showed the possibility of classifying maize seeds varieties. The important wavelengths between 1110 nm and 1380 nm might be attributed to the second overtone of C–H stretch.^38^ The spectral band at 1405 nm might be attributed to the O–H stretch.^39^ The spectral bands at 1460 nm and 1470 nm might be attribute to the first overtone of N–H stretching.^40^ The spectral bands at 1564 nm, 1588 nm and 1625 nm might be attributed to the N–H stretching.^41^

**Table 1:** Important wavelengths recognized by PCA

Number	Important wavelengths (nm)
19	1001, 1025, 1116, 1132, 1156, 1180, 1204, 1220, 1254, 1289, 1301, 1351, 1375, 1405, 1460, 1470, 1564, 1588, 1625

### 3.3 Classification models

The object-wise spectra of each single seed sample were extracted. SVM and RBFNN classification models were established based on object-wise spectra. First, the eight varieties of maize seeds were classified. Second, common maize seeds and silage maize seeds were classified, and four varieties of common maize seeds and four varieties of silage maize seeds were classified, respectively.

#### 3.3.1 Classification of eight varieties of maize seeds

The eight varieties of maize seeds were randomly divided into the calibration and prediction sets at a ratio of 2 : 1 to establish the classification models. For the classification of eight varieties of maize seeds, the penalty parameter (*c*) of the SVM model was 256 and the kernel function (*g*) parameter was 0.5. The accuracy of the SVM calibration set and prediction set were 87.10% and 86.87%, respectively. To explore the classification results of eight varieties of maize seeds, Table 2 shows the confusion matrix of SVM model for classification of eight varieties of maize seeds. As shown in Table 2, except the Variety DT397, all the varieties were well classified with the accuracy of calibration set and prediction set over 91% and 92%, respectively. Most of the Variety DT397 were misclassified as Variety QC29, and a small part was misclassified as Variety QC8, which was the main reason for the low classification accuracy of the eight varieties of maize seeds.

**Table 2:** Confusion matrix of SVM model for classification of eight varieties of maize seeds

		DT397	QC8	QC11	QC13	QC9	QC19	QC29	QC513	Accuracy (%)
Calibration	DT397	735	206	0	0	1	1	2457	0	21.62
	QC8	233	3099	0	0	27	3	38	0	91.15
	QC11	4	0	3306	4	0	49	0	37	97.24
	QC13	0	0	0	3378	7	1	10	4	99.35
	QC9	0	18	0	45	3333	0	4	0	98.03
	QC19	2	46	30	2	0	3284	0	36	96.59
	QC29	88	54	3	24	2	7	3222	0	94.76
	QC513	0	0	10	2	0	54	0	3334	98.06
Prediction	DT397	369	107	0	0	0	1	1223	0	21.71
	QC8	95	1572	0	0	11	0	22	0	92.47
	QC11	2	0	1644	2	0	22	1	29	96.71
	QC13	0	0	4	1683	2	1	5	5	99.00
	QC9	0	7	0	29	1659	0	5	0	97.59
	QC19	0	23	11	1	0	1643	1	21	96.65
	QC29	37	36	3	19	2	3	1600	0	94.12
	QC513	0	0	12	1	1	42	0	1644	96.71

The RBFNN model for the classification of eight maize seeds were established. The spread rate (*s*) of the RBFNN model was 8.9. The accuracy of the RBFNN calibration set and prediction set were both 88.41%. To explore the classification results of eight varieties of maize seeds, Table 3 shows the confusion matrix of RBFNN model for classification of eight maize seeds. As shown in Table 3, except the Variety DT397 and QC29, all the varieties were well classified with the accuracy of calibration set and prediction set over 97% and 96%, respectively. In the Variety DT397, most of them were misclassified with the accuracy of calibration and prediction only about 37%. Most of Variety DT397 were misclassified as Variety QC29, and a small part was misclassified as Variety QC8. In the Variety QC29, a small part was misclassified, most of which were misclassified as Variety DT397, and a small part was misclassified as other varieties such as QC8, QC13, and QC19.

**Table 3:** Confusion matrix of RBFNN model for classification of eight varieties of maize seeds

		DT397	QC8	QC11	QC13	QC9	QC19	QC29	QC513	Accuracy (%)
Calibration	DT397	1290	165	1	0	0	0	1943	1	37.94
	QC8	69	3317	3	0	11	0	0	0	97.56
	QC11	0	7	3384	1	0	4	0	4	99.53
	QC13	0	0	2	3391	1	2	3	1	99.74
	QC9	0	10	2	28	3360	0	0	0	98.82
	QC19	0	11	3	1	0	3366	0	19	99.00
	QC29	647	48	3	80	0	25	2593	4	76.26
	QC513	0	0	2	1	0	50	0	3347	98.44
Prediction	DT397	639	110	3	1	3	0	943	1	37.59
	QC8	46	1646	2	0	5	0	0	1	96.82
	QC11	0	3	1687	0	2	3	1	4	99.24
	QC13	0	1	3	1693	1	0	0	2	99.59
	QC9	0	6	3	16	1673	0	2	0	98.41
	QC19	1	7	2	0	0	1675	0	15	98.53
	QC29	331	54	2	44	3	6	1257	3	73.94
	QC513	0	0	3	0	1	38	0	1658	97.53

From Tables 2 and 3, SVM and RBFNN models had the misclassification of Variety DT397 and Variety QC29. Variety DT397 was misclassified as Variety QC29 in SVM model, which also existed in RBFNN model. Besides, Variety QC29 was misclassified as Variety DT397 and other varieties in RBFNN model. Overall, the classification accuracy of RBFNN model was good compared with that of SVM. For different models, the classification of eight varieties of maize seeds has similar results. Different varieties of maize seeds have an impact on classification.

The maize seeds of Variety DT397 is common maize seeds and the Variety QC29 is silage maize seeds. To explore the influence of varieties on classification results, the classification of common maize seeds and silage maize seeds based on RBFNN model was studied.

#### 3.3.2 Classification of common maize seeds and silage maize seeds

Four varieties of common maize seeds were considered as one class, and four varieties of silage maize seeds were considered as another class. The common maize seeds and silage maize seeds were randomly divided into the calibration and prediction sets at a ratio of 2 : 1 to establish the classification models. The accuracy of the SVM calibration set and prediction set were 87.88% and 87.23%, respectively, with the *c* of the SVM model was 256 and the *g* was 4. Table 4 shows the confusion matrix of SVM model for classification of common maize seeds and silage maize seeds. As shown in Table 4, the accuracy of calibration set and prediction set for the classification of common maize seeds were slightly higher than that of silage maize seeds. The accuracy of calibration set and prediction set for the classification of common maize seeds in SVM model were higher than 88%, and the accuracy of calibration set and prediction set for the classification of silage maize seeds were higher than 85%.

**Table 4:** Confusion matrix of SVM model and RBFNN model for classification of common maize seeds and silage maize seeds

Model			Common^a^	Silage^a^	Accuracy (%)
SVM	Calibration	Common^a^	12 156	1444	89.38
		Silage^b^	1853	11 747	86.38
	Prediction	Common^a^	6025	775	88.60
		Silage^b^	962	5838	85.85
RBFNN	Calibration	Common^a^	12 120	1480	89.12
		Silage^b^	1554	12 046	88.57
	Prediction	Common^a^	6022	778	88.56
		Silage^b^	777	6023	88.57

a Common maize seeds.

b Silage maize seeds.

There might be similarities in appearance and composition among different varieties of common maize seeds and different varieties of silage maize seeds, causing confusion in the classification process.

The RBFNN model was used to classify the common maize seeds and silage maize seeds, with the *s* of the RBFNN model was 8.5. The accuracy of the RBFNN calibration set and prediction set were both 88.41%. Table 6 shows the confusion matrix of RBFNN model for classification of common maize seeds and silage maize seeds. Misclassification was existed between the common maize seeds and silage maize seeds. As shown in Table 6, the accuracy of calibration set and prediction set for the classification of common maize seeds in RBFNN model were higher than 88%, and the accuracy of calibration set and prediction set for the classification of silage maize seeds were both 88.57%.

**Table 5:** Confusion matrix of SVM model and RBFNN model for classification of four varieties of common maize seeds

Model			DT397	QC8	QC11	QC13	Accuracy (%)
SVM	Calibration	DT397	3226	174	0	0	94.88
		QC8	167	3233	0	0	95.09
		QC11	2	0	3397	1	99.91
		QC13	0	0	1	3399	99.97
	Prediction	DT397	1605	95	0	0	94.41
		QC8	81	1618	1	0	95.18
		QC11	1	0	1695	4	99.71
		QC13	0	0	5	1695	99.71
RBFNN	Calibration	DT397	3296	104	0	0	96.94
		QC8	92	3306	0	2	97.24
		QC11	0	0	3398	2	99.94
		QC13	0	1	4	3395	99.85
	Prediction	DT397	1631	67	1	1	95.94
		QC8	57	1642	0	1	96.59
		QC11	0	1	1699	0	99.94
		QC13	0	0	2	1698	99.88

**Table 6:** Confusion matrix of SVM model and RBFNN model for classification of four varieties of silage maize seeds

Model			QC9	QC19	QC29	QC513	Accuracy (%)
SVM	Calibration	QC9	3397	0	3	0	99.91
		QC19	0	3366	1	33	99.00
		QC29	2	6	3391	1	99.74
		QC513	0	35	0	3365	98.97
	Prediction	QC9	1692	0	8	0	99.53
		QC19	5	1670	2	23	98.24
		QC29	11	2	1687	0	99.24
		QC513	3	37	0	1660	97.65
RBFNN	Calibration	QC9	3397	1	2	0	99.91
		QC19	0	3386	0	14	99.59
		QC29	1	13	3382	4	99.47
		QC513	0	27	0	3373	99.21
	Prediction	QC9	1698	0	2	0	99.35
		QC19	1	1688	1	10	99.29
		QC29	6	4	1688	2	99.29
		QC513	0	35	0	1665	97.94

The comparison between the classification results of SVM model and RBFNN model could be seen in Table 4. There existed misclassification between different varieties of maize seeds. SVM model and RBFNN model had the similar results, and the classification accuracy of RBFNN model were slightly higher than that of SVM model.

#### 3.3.3 Classification of four varieties of common maize seeds and four varieties of silage maize seeds

Considering the influence of maize varieties in the process of classification, the classification of four varieties of common maize seeds and four varieties of silage maize seeds was studied respectively. Four varieties of common maize seeds and four varieties of silage maize seeds were randomly divided into the calibration and prediction sets at a ratio of 2 : 1 to establish the classification models.

For the classification of four varieties of common maize seeds, the *c* of the SVM model was 256 and the *g* was 2. The accuracy of the SVM calibration set and prediction set were 97.46% and 97.25%, respectively. The *s* of the RBFNN model was 22.8. The accuracy of the RBFNN calibration set and prediction set were 98.49% and 98.09%, respectively. Table 5 shows the confusion matrix of SVM model and RBFNN model for the classification of four varieties of common maize seeds. As shown in Table 5, the SVM model and RBFNN model had similar results. Variety DT397 and Variety QC8 were likely to be misclassified. Variety QC11 and Variety QC13 were well classified with the accuracy higher than 99%. The classification accuracy of RBFNN model were slightly higher than that of SVM model.

The accuracy of the SVM calibration set and prediction set were 99.40% and 98.66%, respectively. The *s* of the RBFNN model was 12.8. The accuracy of the RBFNN calibration set and prediction set were 98.49% and 98.09%, respectively. Table 6 shows the confusion matrix of SVM model and RBFNN model for the classification of four varieties of silage maize seeds. As shown in Table 6, the SVM model and RBFNN model had similar results. All varieties of silage maize seeds were well classified, with the accuracy of about 99%.

From Tables 5 and 6, SVM model and RBFNN model showed similar performances for the classification of four varieties of common maize seeds and the classification of four varieties of silage maize seeds. Four varieties of silage maize seeds could be well classified. In the classification of four varieties of common maize seeds, Variety DT397 and Variety QC8 had obvious misclassification, but had little effect on the overall classification accuracy. SVM model and RBFNN model could be used to classify different varieties of maize seeds. Different classification models showed similar results for the classification of different varieties of maize seeds, confirming the identifiability among different varieties of maize seeds.

## 4. Conclusions

The classification of maize seeds of different varieties based NIR-HSI was studied. The classification of seeds of silage maize and common maize was involved, and the classification of the seeds into four varieties of silage maize and four varieties common maize respectively were also involved. Maize seeds pixel-wise spectra were extracted to conduct PCA analysis and form the PCA scores images. The scores images of the first six PCs indicated the difference among different varieties of maize seeds. Based on the extracted object-wise spectrum of each single seed sample, the SVM and RBFNN classification models were established, and satisfactory classification results were obtained. For the classification of eight varieties of maize seeds, the prediction accuracy of the SVM model and RBFNN model were 86.87% and 88.41%, respectively. For the classification of common maize seed and silage maize seeds, the prediction accuracy of the SVM model and RBFNN model were 88.23% and 88.41%, respectively. For the classification of four varieties of common maize seeds, the prediction accuracy of the SVM model and RBFNN model were 97.25% and 98.09%, respectively. For the classification of four varieties of silage maize seeds, the prediction accuracy of the SVM model and RBFNN model were 98.66% and 99.10%, respectively.

The classification of maize seeds of different varieties based on NIR-HSI was feasible. The classification of common maize seeds and silage maize seeds and the classification of different varieties of silage maize seeds based on NIR-HSI could be achieved. The approach did not require complicate sample pretreatment. It was fast and convenient. In the future, the varieties and the number of samples should be increased to establish a maize seeds classification library, which is more convenient for rapid classification of maize seeds.

## Conflicts of interest

There are no conflicts to declare.

## Supplementary Material

RA-010-C9RA11047J-s001

RA-010-C9RA11047J-s002

RA-010-C9RA11047J-s003

RA-010-C9RA11047J-s004

RA-010-C9RA11047J-s005

RA-010-C9RA11047J-s006

RA-010-C9RA11047J-s007

RA-010-C9RA11047J-s008

RA-010-C9RA11047J-s009

RA-010-C9RA11047J-s010

RA-010-C9RA11047J-s011

RA-010-C9RA11047J-s012
